# Targeted Brain Derived Neurotropic Factors (BDNF) Delivery across the Blood-Brain Barrier for Neuro-Protection Using Magnetic Nano Carriers: An *In-Vitro* Study

**DOI:** 10.1371/journal.pone.0062241

**Published:** 2013-04-30

**Authors:** Sudheesh Pilakka-Kanthikeel, Venkata Subba Rao Atluri, Vidya Sagar, Shailendra K. Saxena, Madhavan Nair

**Affiliations:** 1 Department of Immunology, Institute of NeuroImmune Pharmacology, Herbert Wertheim College of Medicine, Florida International University, Miami, Florida, United States of America; 2 CSIR-Centre for Cellular and Molecular Biology, Uppal Road, Hyderabad, India; University of Nebraska Medical Center, United States of America

## Abstract

Parenteral use of drugs; such as opiates exert immunomodulatory effects and serve as a cofactor in the progression of HIV-1 infection, thereby potentiating HIV related neurotoxicity ultimately leading to progression of NeuroAIDS. Morphine exposure is known to induce apoptosis, down regulate cAMP response element-binding (CREB) expression and decrease in dendritic branching and spine density in cultured cells. Use of neuroprotective agent; brain derived neurotropic factor (BDNF), which protects neurons against these effects, could be of therapeutic benefit in the treatment of opiate addiction. Previous studies have shown that BDNF was not transported through the blood brain barrier (BBB) *in-vivo.*; and hence it is not effective *in-vivo*. Therefore development of a drug delivery system that can cross BBB may have significant therapeutic advantage. In the present study, we hypothesized that magnetically guided nanocarrier may provide a viable approach for targeting BDNF across the BBB. We developed a magnetic nanoparticle (MNP) based carrier bound to BDNF and evaluated its efficacy and ability to transmigrate across the BBB using an *in-vitro* BBB model. The end point determinations of BDNF that crossed BBB were apoptosis, CREB expression and dendritic spine density measurement. We found that transmigrated BDNF was effective in suppressing the morphine induced apoptosis, inducing CREB expression and restoring the spine density. Our results suggest that the developed nanocarrier will provide a potential therapeutic approach to treat opiate addiction, protect neurotoxicity and synaptic density degeneration.

## Introduction

Drug abuse is one of the major concerns of present United States. Drugs of abuse such as opiates, cocaine, methamphetamine are frequently being used by individuals infected with HIV-1 [1]. Epidemiological data demonstrate that opioid abuse is a risk factor for HIV-1 infection and progression to AIDS and other neurodegenerative changes [2]. In recent years, the incidence of HIV-1 infection has increased in drug abusing populations [3]. HIV and most abused drugs (morphine, heroine, etc) target areas in brain such as basal ganglia and cortex that are rich in opioidergic receptors [4].

Though opiates, especially morphine and heroin are known to exert their effects through μ-opiate receptor, the exact mechanism by which opiates act as a cofactor for HIV infection is not clear. However, it is reported that μ opioid ligands act synergistically with HIV proteins (tat; transactivator and gp120) to increase the receptors necessary for the transmission of the virus and potentiate the HIV-related neurotoxicity [5]. Opiates also have been shown to induce apoptosis of neuronal cells, microgila, macrophages and monocytes. Apoptosis of neuronal cells, involved in brain cell death, accompany neurodegenerative disorders, such as Alzheimer’s disease and Parkinson’s disease [6], [7], [8]. Therefore, the need to protect neuronal cells against the toxic effect of drugs of abuse using neuroprotective agents is of therapeutic importance.

Many of the drugs aimed at treating different CNS related diseases are not very effective to do so in the brain because of the impenetrability of these drugs across blood brain barrier (BBB) [9]. The BBB is a major physiological barrier that restricts the transport of most small hydrophilic molecules and macromolecules from the cerebrovascular circulation into the brain. The selective permeability of the BBB is due to the distinct morphology and enzymatic properties of endothelial cells that enable them to form complex tight junctions with minimal endocytic activity. This provides a physiological barrier that limits the transport of many blood-borne elements such as macromolecules and circulating leukocytes to the brain [10], [11].

Brain derived neurotropic factor (BDNF), a member of neurotrophic factor family is one of the most powerful neuroprotective agents for those neurons that degenerate in HIV associated Dementia (HAD) [12], [13], [14]. BDNF has been effective in preventing gp120-mediated toxicity in *in-vitro* and *in-vivo* conditions [15], [16], increasing survival of dopaminergic neurons of the substantia nigra after 6-OH-dopamine or 1-methyl-4-phenyl-1,2,3,6 tetrahydropyridine (MPTP) lesions [17], [18], protecting serotonergic neurons against the neurotoxin *p-*chloroamphetamine [19], and rescues cortical neurons from stroke-mediated apoptotic cell death [20]. These results provide the rationale for using BDNF experimentally to rescue neurons, promote regeneration of synaptic connections and enhance neuronal plasticity in the brains of drug abusers. Therefore, use of a neuroprotective agent such as BDNF in addition to a μ-opioid receptor antagonist could be of therapeutic benefit in the treatment of opiate addiction [14]. However, the major problem with options using BDNF is its inability to cross the BBB [21], [22] and hence the transport of drugs across BBB remains a challenge.

In recent years, advent of nanotechnology has stimulated the development of innovative systems for the delivery of drugs and diagnostic agents [23]. A significant research exploring nanocarrier drug delivery systems for crossing the BBB has focused on the delivery of anticancer drugs to brain tumors. More recently, the magnetic nanoparticles have attracted significant importance in biomedical applications and have been increasingly used as carrier for binding proteins, enzymes, or drugs. We and others have also previously reported successful immobilization of several clinically and biotechnologically important proteins and enzymes onto magnetic nanoparticles [24]. In the present study, we hypothesized that by binding to a magnetically guided nanocarrier, the impermeability of BBB by free BDNF can be overcome. We carried out the binding of BDNF with magnetic nano particle (MNP) and studied the efficacy and ability to cross BBB.

## Materials and Methods

### Ethics Statement

Buffy coat was purchased from the community blood bank, for which ethics committee approval is not needed. However, the NIH grant which funded this work has institutional IRB approval (#013009-00).

### Preparation of Magnetic Nanoparticles

Magnetic nanoparticles were prepared by coprecipitating of Fe^2+^ and Fe^3+^ ions in alkaline solution and treating under hydrothermal condition as described earlier [25]. Hundred millilitres solution of 1 M FeSO_4_·7H_2_O and 2M FeCl_3_ (Sigma) were thoroughly mixed and added to 8 M ammonium hydroxide (Sigma) with constant stirring at 25°C. The resultant black magnetite particles were washed repeatedly with hot distilled water to remove impurity ions such as chlorides and sulphates and dispersed in Tris-EDTA buffer (pH 7.5). The yield of precipitated magnetic nanoparticles was determined by removing known aliquots of the suspension and drying to a constant mass in an oven at 60°C. Finally, the particles were dispersed in TE buffer at a suspension concentration of 10 mg/ml. The particles were characterized for size using transmission electron microscopy (TEM).

### Binding of BDNF with Magnetic Nanoparticles

For the binding experiment, different ratios of magnetic nanoparticles and BDNF (1∶0.05, 1∶0.01, 1∶0.015, 1∶0.02, 1∶0.025, 1∶0.03, 1∶0.35) were mixed in TE buffer pH 7.5, followed by incubating the mixture on a shaker (100 rpm) for 3 hrs at room temperature. After incubation, the magnetic particles bound with BDNF were attracted by application of an external magnetic field. The supernatant containing the unbound BDNF was collected and the pellet was resuspended in appropriate volume of TE buffer pH 7.5 and stored at 2°C to 8°C until further use.

### BDNF ELISA

The binding efficiency (µg BDNF/mg of magnetic nanoparticles) was determined by measuring the amount of BDNF in the unbound fraction by ELISA (BDNF Kit from R&D Systems, Minneapolis, MN, USA) as per manufacturer’s recommendation. The amount of BDNF bound to the magnetic nanoparticles was calculated from the difference between the total BDNF added and unbound BDNF measured in the supernatant.

### Preparation of Peripheral Blood Mononuclear Cells (PBMC)

PBMCs were isolated from buffy coat, procured from the community blood bank, by standard, density gradient centrifugation as previously described [26], [27]. Briefly, leukopack was diluted by adding five volumes of phosphate-buffered saline (PBS) and overlaid over histopaque (Sigma Aldrich, St. Louis, MO). The samples were centrifuged at 1200 g for 20 min at room temperature. PBMCs were carefully retrieved from the interface, washed twice with PBS and resuspended in RPMI-1640.

### Apoptosis Measurement

PBMC (1X10^6^ cells/ml) were treated with morphine (10^−7^ M) for 48 hrs. Cells were treated with free BDNF and MNP-BDNF (BDNF dose; 10, 50 & 100 ng/ml) 12 hr before morphine treatment. At the termination of morphine treatment, cells were washed twice with cold PBS and then resuspended in 1X binding buffer at a concentration of 1×10^6^ cells/ml. 100 µl from this is added to a 5 ml FACS tubes, followed by incubation with 5 µl each of Annexin V and 7-AAD (BD Biosciences) for 15 minutes at RT in the dark. After incubation, 400 µl of 1X binding buffer is added to each tube, mixed gently and analyzed by FACScalibur within 1 hr. The untreated cells, which served as control, is used for defining the basal level of apoptotic and dead cells. The percentage of cells that have been induced to undergo apoptosis is then determined by subtracting the percentage of apoptotic cells in the untreated population from percentage of apoptotic cells in the treated population. Cells treated with camptothecin for 5 hrs at 37°C is used as positive control.

### Toxicity

Live/Dead dye (ViViD; Invitrogen) was resuspended in DMSO as directed by kit and diluted 1∶1,000 in FACS buffer. PBMC treated MNP-BDNF and MNP were washed with 1X with FACS buffer and incubated for 15 minutes with 50 µl of diluted dead cell discrimination dye on ice in the dark. Cells were then washed 2X with FACS buffer and analyzed by flowcytometry.

### In-vitro BBB Preparation

The BBB model was established according to the procedure described earlier [28]. The BBB model consisted of 2-compartment wells in a culture plate with the upper compartment separated from the lower by a cyclopore polyethylene terephthalate membrane (Collaborative Biochemical Products, Becton Dickinson, San Jose, CA) with a pore diameter of 3 µm. In a 24-well cell culture insert (surface area 0.3 cm 2), primary human brain microvascular endothelial cells (BMVEC; 1 × 10^5^) were grown to confluency on the upper side while a confluent layer of human astrocytes (AM) were grown on the underside. BMVEC and AM were procured from Sciencell Research Laboratories, Carlsbad, CA. Intactness of the BBB was judged by measuring the trans endothelial electrical resistance (TEER) using Millicell ERS microelectrodes (Millipore) after 6 days of culture. A mean TEER value of 150 to 200 ohms/cm^2^ cell culture insert is consistent with the formation of the BBB. The BBB model was used for experiments at least 6 days after cell seeding.

### CREB

Total RNA was isolated using RNAeasy kit (Qiagen) as per the manufacturer’s protocol. Total RNA (100 ng) was converted into cDNA. CREB mRNA was measured by real-time quantitative reverse transcription-polymerase chain reaction (RT-PCR) using SYBR green master mix from Stratagene (La Jolla, CA) using the Stratagene 3000 instrument that detects and plots the increase in fluorescence versus PCR cycle number producing a continuous measure of PCR amplification. Relative expression of mRNA species was calculated using the comparative C_T_ method [29]. All data were controlled for quantity of RNA input by performing measurements on an endogenous reference gene, β-actin. In addition, results on RNA from treated samples were normalized to results obtained on RNA from the control, untreated sample.

### DiI staining for Measurement of Spine Density and Size

Human neuroblastoma SK-N-MC cells were obtained from ATCC. SK-N-MC cells were grown in Eagle’s minimal essential medium (MEM) containing 10% fetal bovine serum (FBS), 5 mM sodiumpyruvate, 100 units/ml penicillin, and 100 mg/ml streptomycin at 37°C with 5% CO2. SK-N-MC cells were grown onto 22 mm×50 mm glass coverslips placed in a petri-dish. Cells were treated with morphine for 48 hrs. Cells were treated in respective wells with either free BDNF or MNP-BDNF for 12 hrs before morphine treatment. Cells were fixed in 4% Formaldehyde in PBS for 30 min at room temperature. The fluorescent membrane tracer 1,1′-Dioctadecyl-3,3,3′,3′-tetramethylindocarbocyanine perchlorate (DiI; 5 µg/ml in PBS) was directly added onto the fixed cultures and allowed to incubate for 60–90 min at room temperature. The coverslips were placed at 4°C in small petri dishes containing PBS and allowed for 6–12 hr for transport of the dye before confocal microscopy.

### Confocal Microscopy

Confocal images were obtained using TCS SP2 confocal laser scanning microscope (Leica Microsystems, Germany) at 488 nm (100%) illusion of an argon-ion laser using 60× oil immersion objectives with high numeric aperture and 2.5× confocal electronic zoom settings to visualize individual cells and dendrites. Twenty Optical serial sections of 0.14 µm/section (2.8 µm total) through the cells and reconstructed to yield complete “three dimensional” images of individual cells in focus. ImageJ software program was used to quantify DiI-labeled cells. Dendritic segments were chosen randomly from the apical and basal regions and at least one soma’s length away from the cell soma. The spine density is defined as the number of spines per unit length [30]. The length of randomly selected dendritic segment was measured, the number of spines along that length was counted and spine density was calculated by dividing the total number of spines by dendrite length, as expressed as spines/10 µm.

### Statistical Analysis

Data were expressed as mean±SEM of triplicate values for each experiment. Student’s t-test was used to compare means of two groups using Prism (GraphPad; San Diego, CA). P<0.05 was considered significant.

## Results

**Figure 1:**
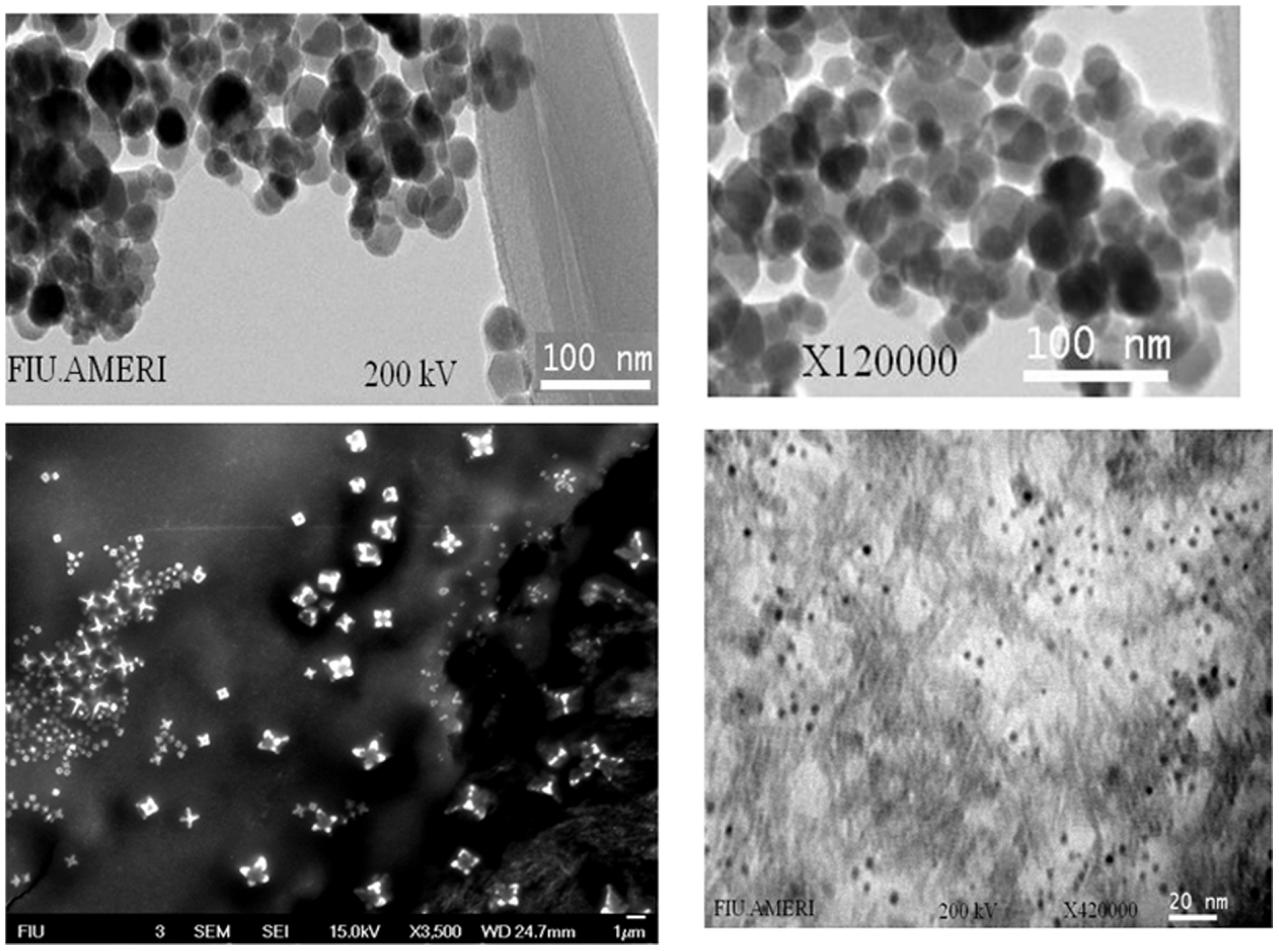
TEM micrograph of magnetic nano particles. The average particle size is ∼ 60 nm.

TEM imaging of MNP was performed to confirm the particle size, obtain the distribution and observe general morphology of the particles. TEM examination of prepared MNP revealed the mean particle size to be 60 nm (figure 1). The particles were uniform in distribution and mostly spherical in shape.

**Figure 2:**
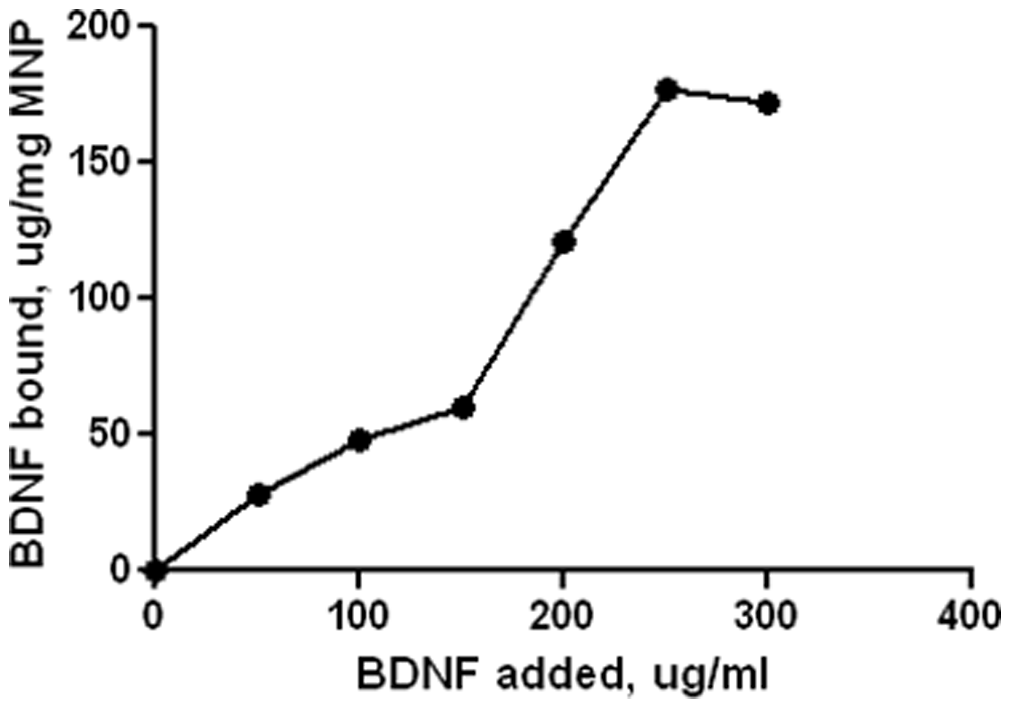
Binding isotherm for BDNF on MNP. Ratios of 1∶0.05, 1∶0.01, 1∶0.015, 1∶0.02, 1∶0.025, 1∶0.03, 1∶0.35 of MNP and BDNF were used for 3 hrs. Binding efficiency (µg BDNF/mg MNP) was calculated by ELISA.

After the characterization of MNP is done, we prepared a formulation of MNP carrying BDNF. Efficiency of BDNF binding to MNP was calculated by using different ratios of MNP and BDNF (1∶0.05, 1∶0.01, 1∶0.015, 1∶0.02, 1∶0.025, 1∶0.03, 1∶0.35) in TE buffer. Data given on figure 2 represents the binding isotherm of BDNF binding to MNP. The binding efficiency (µg BDNF/mg of magnetic nanoparticles) was determined by measuring the amount of BDNF in the unbound fraction by ELISA. The difference between the total BDNF added and unbound BDNF in the supernatant was used to calculate the amount of BDNF bound to the magnetic nanoparticles. The binding efficiency was in the range of 177 µg of BDNF bound per mg of MNP in 3 hours, which correspond to approximately 70% binding of BDNF to MNP. This shows that BDNF was effectively bound to MNP.

**Figure 3:**
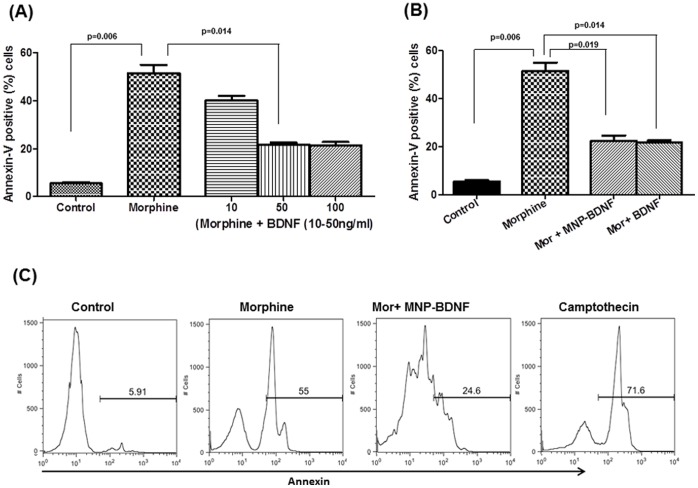
MNP bound BDNF is effective in suppressing the apoptosis induced by morphine. (A) Morphine induces apoptosis of human PBMCs. PBMCs were cultured alone or with 10^−7^ M concentration of morphine for 48 hrs. Free and bound BDNF (10, 50,100 ng/ml) were added to respective cultures 24 hr before morphine treatment. After treatment, cells are stained with Annexin-V and 7-AAD analyzed by FACScalibur within 1 hr. The untreated cells, which served as control, is used for defining the basal level of apoptotic and dead cells. (B) Representative histogram showing Annexin-V expression in treated cells.

After the binding efficiency was calculated, further experiments were carried out to check whether BDNF activity is retained after immobilization by direct binding to MNP. Our aim was to evaluate and compare if the efficiency of bound BDNF is similar to free BDNF. Since BDNF has been shown to prevent apoptosis in different cells including brain cells [31], we wanted to check the efficiency of nanofurmulation by evaluating its ability to suppress apoptosis induced by morphinein PBMC. As expected, morphine (10^−7^M) induced apoptosis (55%) in PBMC compared to untreated cells (p = 0.006) as shown in figure 3A. We saw that BDNF pretreatment could suppress morphine induced apoptosis in dose dependent fashion; optimum response being at 50 ng/ml (p = 0.019). Based on this response, for our further experiments, we used 50 ng/ml concentration of BDNF. Next, we wanted to evaluate if MNP-BDNF formulation is as effective as free BDNF and can revert the morphine induced apoptosis. The results showed that BDNF bound to MNP also reversed the morphine induced effect (p = 0.014; figure 3B), suggesting that MNP-BDNF was equally effective as free BDNF in suppressing apoptosis, which supports the hypothesis that binding of MNP did not interfere with the enzymatic activity of BDNF.

**Figure 4:**
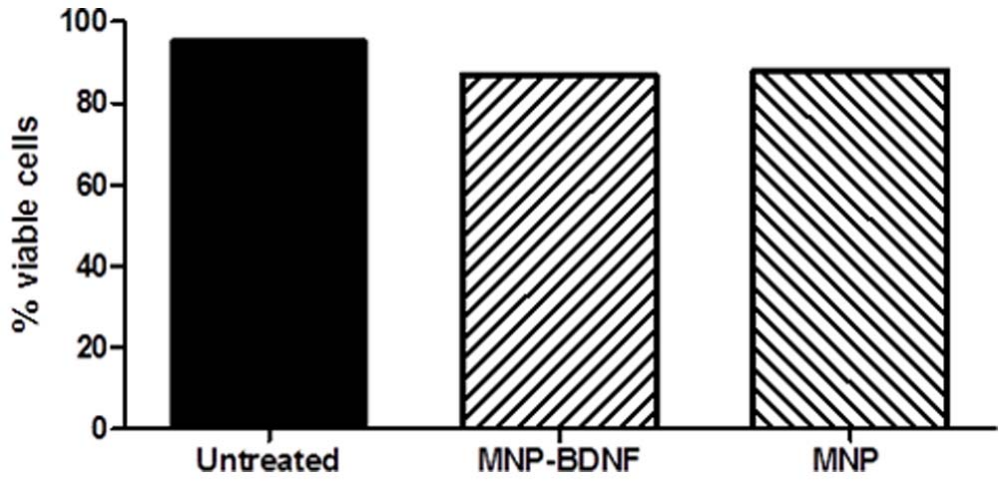
MNP is not toxic to the PBMC. PBMC (1X10^6^/ml) treated with free and bound BDNF were stained with live-dead cell discrimination dye ViViD and analyzed by flowcytometry.

Before proceeding into further experiments, we wanted to make sure that the MNP or MNP-BDNF formulation is not toxic to the cells, and evaluated the cytotoxicity potential of the nanoformulation by flow cytometry using live/dead fixable dead cell stain, ViViD. It was clear from results that either of them did not result in a significant decrease in the viability of cells. Approximately, 80–90% of cells were live (Figure 4), indicating that either MNP alone or MNP-BDNF was not toxic to cells.

**Figure 5:**
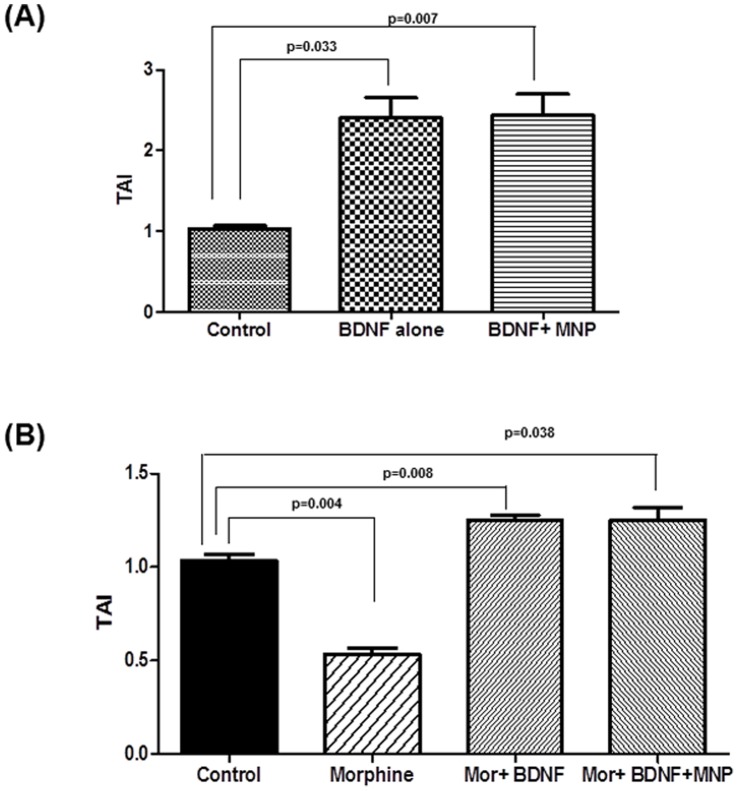
MNP bound BDNF reverses morphine induced CREB downredulation in astrocytes. (A) Morphine significantly downregulated CREB gene expression. Astrocytes (1X10^6^/ml) were cultured with or without morphine (10^−7^ M) for 48 hrs; RNA was extracted, reverse transcribed, cDNA amplified, and gene expression of CREB was determined by real-time quantitative PCR. These data are the mean ± SD of 3 separate experiments. (B) Astrocytes (3X10^6^/ml) were cultured with free and bound BDNF 24 hr before morphine (10^−7^ M) treatment. RNA was extracted, reverse transcribed, cDNA amplified, and gene expression of CREB was determined by real-time quantitative PCR. These data are the mean ± SD of 3 separate experiments.

In order to reconfirm the effectiveness of MNP-BDNF, we carried out additional experiment to check the effect of MNP-BDNF on CREB expression in astrocytes. It has been previously shown that BDNF augments CREB expression [32], which is known to be a player in memory. CREB functions in long-term neuronal plasticity and enhances memory consolidation. We checked the CREB expression in astrocytes treated with BDNF. In agreement with previous reports [32], we found a significant increase in CREB expression with BDNF (TAI = 2.4±0.25, p = 0.033) as shown in figure 5A. Similar to free BDNF, MNP bound BDNF also induced significant CREB expression in astrocytes in (TAI = 2.43±0.27; p = 0.007). Expression level of CREB was comparable with free and MNP bound BDNF, which again supports our previous finding that free and bound BDNF have comparable efficacy.

When astrocytes were treated with morphine, it resulted in significant down regulation of CREB (TAI = 0.533±0.03; p = 0.0004; Figure 5B), which is also in consistent with previous reports [29]. Further, we treated astrocytes with free or bound BDNF 12 hrs before treatment with morphine and checked CREB expression. We found that treatment with BDNF resulted in reversal of morphine induced down regulation of CREB. Moreover, similar to the previous experiment, both free (TAI = 1.25±0.03; p = 0.008) and bound BDNF (TAI = 1.253±0.06; p = 0.03) were effective in reversing morphine induced down regulation of CREB respectively.

**Figure 6:**
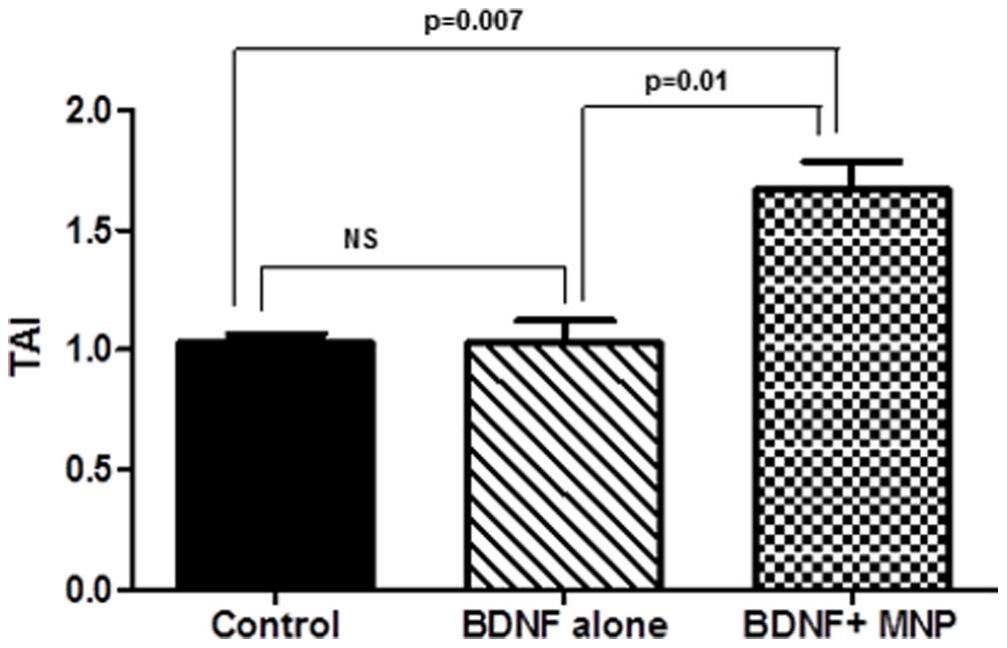
MNP bound BDNF efficiently crosses BBB and induces CREB expression. Free and bound BDNF were added to the respective wells of BBB, in the upper chamber. A weak magnet was placed on the basal side of BBB. Astrocytes (3X10^6^/ml) were collected from basal side, RNA was extracted, reverse transcribed, cDNA amplified, and gene expression of CREB was determined by real-time quantitative PCR. These data are the mean ± SD of 3 separate experiments.

Since our previous results showed that MNP-BDNF is as effective as free BDNF in its activity, our next aim was to study if MNP bound BDNF is able to cross the BBB, and if it crosses the BBB, whether it retains its efficiency after crossing the membrane. For this, we set up an *in-vitro* BBB model. We placed MNP bound BDNF or free BDNF on the upper chamber of BBB and allowed them to pass through the BBB under the influence of a magnet placed in the basal side and evaluated the expression of CREB on astrocytes on the basal side of BBB. Culture treated with BDNF alone did not induce any change in CREB expression in astrocytes in the basal side. Whereas culture treated with MNP-BDNF showed an upregulation of CREB (TAI = 1.67±0.12; p = 0.007, figure 6). This result confirmed that unlike free BDNF, BDNF bound to MNP is able to cross BBB and is effective in its function.

We also evaluated the amount of BDNF transported across BBB to understand the efficacy of MNP to transport BDNF across BBB. We measured the concentration of BDNF in the basal portion of BBB, and calculated the percentage of BDNF transported through BBB. From the results, it was clear that approximately 73% of the MNP bound BDNF was able to transport across BBB. In addition, to confirm that passage of MNP-BDNF through BBB did not affect the BBB, intactness of membrane was confirmed by TEER reading. It was clear from the TEER reading that passage of MNP-BDNF through BBB did not interfere with integrity of the membrane; 280.4 vs 276.5 (Table 1).

**Table 1:** Transendothelial electrical resistance (TEER) values before and after treatment with MNP-BDNF.

	Before Treatment	After Treatment
Untreated	287.6±9.4	286.67±7.2
MNP-BDNF treatment	280.4±10.4	276.56±9.7

**Figure 7:**
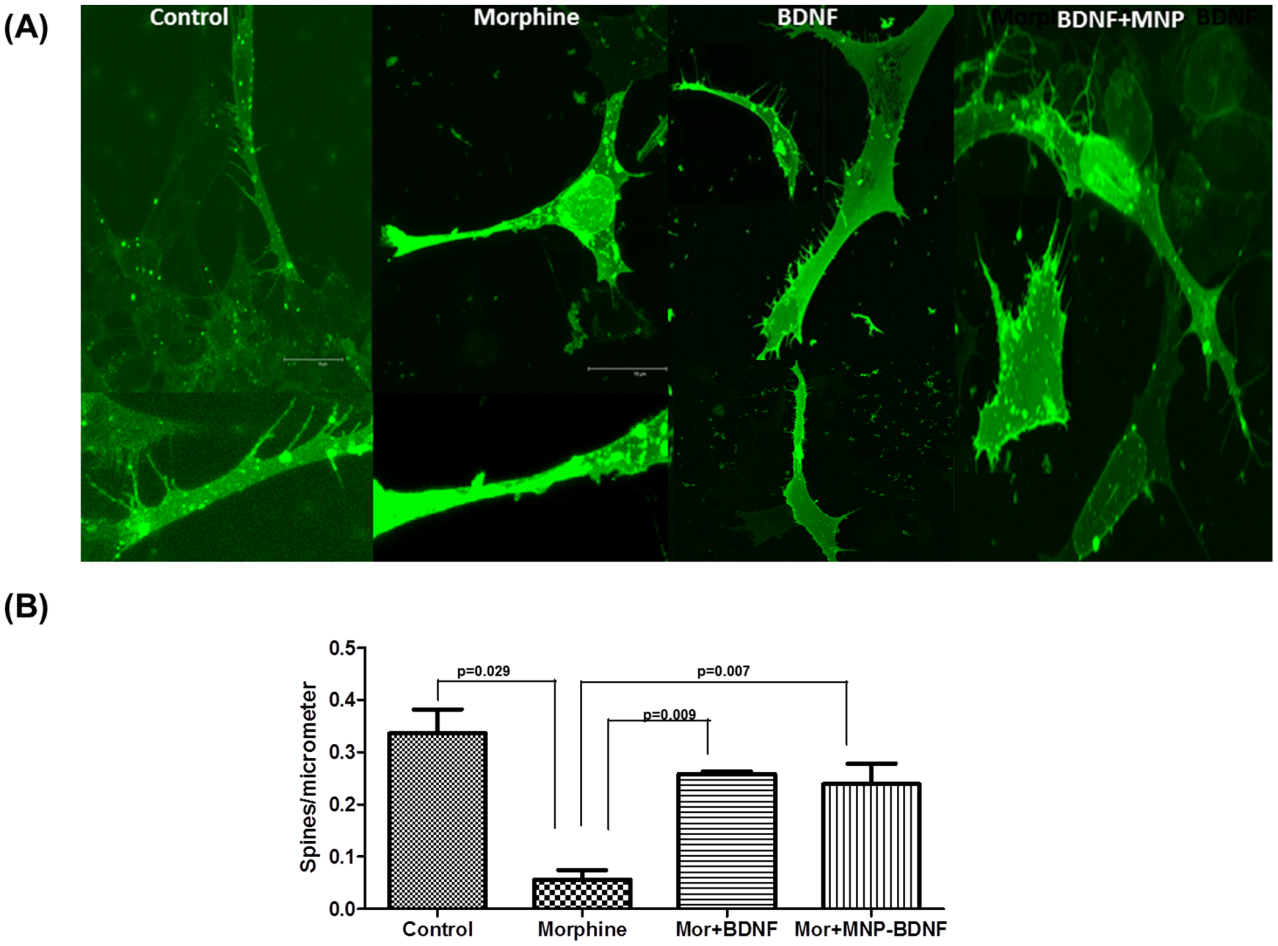
Morphine induced spine density is reversed by MNP bound BDNF. (A) Spine density measurement by Image J analysis. SK-N-MC neuroblastoma cells were grown onto glass coverslips placed in a petridish and treated with BDNF and/or morphine. After treatment, cells were stained with 1, 1′-Dioctadecyl-3, 3,3′,3′-tetramethylindocarbocyanine perchlorate (DiI) and visualized using confocal microscope. Representative image is shown in fig. 7A. (B) Dendritic segments were chosen randomly from the apical and basal regions. The length of randomly selected dendritic segment was measured, the number of spines along that length was counted and spine density was calculated by dividing the total number of spines by dendrite length, as expressed as spines/µm. Figure represents mean of 3 different experiments.

Finally, we tested the ability of our nanoformulation to facilitate the dendrite spine density. Dendritic branching and dendritic spine densities of cells were measured by DiI staining followed by confocal microscopy. Treatment with drugs of abuse has been shown to decrease the dendritic spine density [33]. And BDNF has been reported to increase dendrite numbers [34], [35]. We treated the neuronal cell line SK-N-MC, grown on glass cover slips placed in petridishes, with MNP-BDNF 12 hrs before treatment with morphine and evaluated the dendritic spine density. Treatment of SK-N-MC cells with morphine resulted in significant decrease in total dendrite area (*p<0.00)* and spine density (0.055±0.02; p*<0.002)* compared with untreated control cells (Figure 7). These morphological changes induced by morphine was compensated by MNP-BDNF treatment, which resulted in an increase in dendrite branching compared to morphine treated cells (0.258±0.09; p = 0.009).

## Discussion

Drug abuse is America’s number one enemy. Opiates, especially morphine and heroin are known to exert their effects through µ opiate receptor [4]. Several reports have shown immunomodulation in opiate using individuals [36], [37]. Drugs of abuse act synergistically with HIV proteins to potentiate HIV related neurotoxicity and affects many functions associated with the synaptic plasticity. About one-third of HIV infected population are linked directly or indirectly to injection drug use, with an increase in the incidence of HIV-1 infection in drug abusing populations in recent years [38]. At present, there is no treatment available which alleviate the synergistic effects of opiates and HIV, due to the same fact that therapeutic molecules are unable to cross the BBB.

Brain-derived neurotrophic factor (BDNF), a member of the neurotrophin family of multifunctional neurotrophic factors is capable of regulating neuronal development and survival [39]. BDNF has been shown to prevent caspase-3-mediated apoptosis in cerebellar granule cells in vitro [15] and in the brain in vivo [20]. This finding is especially relevant because the brains of HAD patients show activation of caspase-3 and other pro-apoptotic proteins [40], [41]. Thus, it seems conceivable that BDNF may prevent opiates or gp120-mediated apoptosis and its neurotrophic activity can be extended to neuroprotection. While BDNF exhibits neuroprotective properties in the adult CNS, it affects a variety of events associated with neuronal plasticity during CNS development.

Thus, there is considerable rationale for finding an adjunct therapy to prevent neuronal degeneration and atrophy by taking consideration the neuroprotective potential of BDNF in preventing neurotoxicity. But these large neurotrophic protein molecules do not efficiently cross the blood–barrier into the CNS [42], [43]. BBB restricts the transport of most small hydrophilic molecules and macromolecules from the cerebrovascular circulation into the brain, due to the distinct morphology and enzymatic properties of endothelial cells that enable them to form complex tight junctions with minimal endocytic activity [11], [44], [45]. Several approaches are being pursued to deliver drugs across BBB which include, the use of lipid carrier, tagging drugs to ligands that cross the BBB through a carrier mediated transport (CMT) or receptor mediated transport (e.g. insulin, transferrin, etc) [21]. Some *in-vivo* strategies for delivering neurotrophic factors to the CNS include direct injection into the brain, viral vector upregulation, [46], [47], infusion pump-mediated delivery methods [48], trans-nasal drug delivery, transient disruption of BBB using hyperosomotic solutions and lipidization of small molecules. Unfortunately, these methods presently lack practical relevance for patient treatment and the transport of drugs across BBB still remains a challenge. Clinical trials have demonstrated that systemic deliveries at doses that are sufficiently high to result in therapeutic levels within the CNS parenchyma also result in significant systemic side-effects [49]. In order to solve the BBB drug delivery problems and to comprehend the clinical promise of different neuroprotective factors, new innovative technologies or need for alternative methods of drug delivery are required.

This is where nanotechnology comes into play. A significant research exploring nanocarrier drug delivery systems for crossing the BBB has focused on the delivery of anticancer drugs to brain tumors [50]. More recently, the use of magnetic nanoparticles (MNP) has attracted significant importance in biomedical applications and this has been reported by us and others [51], [52], [53]. MNPs have gained significant attention due to their intrinsic magnetic properties, which enable tracking through the radiology cornerstone, magnetic resonance (MR) imaging. Several *in-vitro* and *in-vivo* studies have been reported with magnetically guided drug targeting systems [54], [55]. However, no attempts have been reported using magnetic nanocarrier drug delivery system to treat drug abuse. The advantage of using magnetic nanoparticles contributes to a precise delivery of drugs to the exact site (e.g. inflammation, cancer etc.) by application of an external magnetic field [56]. Previously our lab have reported ART bound to magnetic nanoparticles were effective in suppressing HIV infection and able to cross BBB [51], [57]. In the present study we aimed to investigate binding of BDNF with MNP followed by functional effectiveness of MNP-BDNF by studying different parameters and its ability to cross BBB.

Apoptosis, a genetically determined mechanism of programmed cell death triggered by a variety of internal and external stimuli, is also a pathologic feature in certain inflammatory diseases of the brain and CNS infections, such as HAD [58]. Opiates have been shown to induce apoptosis of all types of cells including lymphocytes [6], [7], [8], [59], [60], [61], [62] through the activation of caspase-3. These findings are important, considering that inhibitors of caspase-3 activity can rescue neurons from the apoptotic cycle. Neurotrophic factor, BDNF is a class of such inhibitors. Keeping this fact in mind, we checked the efficacy of MNP-BDNF formulation in suppressing the apoptosis induced by morphine in lymphocytes. Our results confirmed that morphine induced apoptosis in PBMC, and this effect was reversed by free BDNF. We also found that MNP bound BDNF was also equally effective as free unbound BDNF in suppressing the apoptosis induced by morphine. This proved that BDNF had efficiently bound to MNP and BDNF is still effective in its bound form.

Activation of CREB, an important factor linking the opioid-regulated secondary messenger systems to alterations in gene expression, is a crucial step during the consolidation of one-trial inhibitory avoidance memory in rats [63], [64], [65], [66], [67]. Opioid receptor stimulation by morphine leads to a decrease in the phosphorylation of CREB [29]. BDNF exerts its role in long-term memory (LTM) formation in the hippocampus, via the activation of CREB in a time-dependent manner [68]. In the next experiment, we studied the ability of MNP-BDNF to modulate the morphine induced CREB expression in astrocytes. As explained previously [29], CREB expression was down regulated after morphine treatment. MNP-BDNF formulation was effective in reversing the morphine effect on CREB expression. This showed that nanoformulation can be used to alleviate morphine induced effects in CNS related problems.

As previously discussed by many, the obstacle in using BDNF is its inability to pass through BBB. So, in our further experiments, we evaluated if MNP-BDNF formulation we prepared can be passed through BBB. For this we used an established in-vitro model of BBB [28], [51] under the influence of an external magnetic field. We saw that under the influence of external magnetic field MNP-BDNF formulation could pass through BBB. Permeability of MNP-BDNF was 3.5 folds higher than free BDNF, suggesting that association with MNP increases the transmigration ability of BDNF across BBB. Magnetic drug targeting of anticancer drugs to treat brain carcinoma has also been reported [52]. In this study, we also showed that MNP-BDNF transmigrated through BBB is also effective in up regulating CREB expression in astrocytes on basal side of the BBB. Free BDNF was less effective, as expected, because of the fact that in its free form BDNF is unable to cross BBB. Approximately 73% of total BDNF added on the top side of BBB were able to cross BBB, when it is present in the bound form with MNP. Moreover transport of MNP-BDNF formulation did not interfere with BBB integrity, as indicated by TEER reading.

Numerous studies have shown that drug addiction alters the function of the neuronal circuit, including changes in neuronal plasticity and synaptic transmitter release [69], [70], [71]. Previous in-vivo studies suggest that morphine administration produces a persistent decrease in dendritic branching and spine density of medium spiny neurons in the nucleus accumbens shell and pyramidal cells in sensory cortex [72], decrease in both total dendrite length and dendritic spine density of neurons in visual cortex [33]. In agreement with these studies, we saw that morphine treatment decreased the spine density and length, which was reversed by MNP-BDNF treatment.

Since one of the major concerns while using nanomaterials in medicine is that of potential toxicity, evaluating cell viability is important for the nanoparticle application in medicine. When we checked the viability of PBMC cultured with MNP and MNP-BDNF by flow cytometry using a dead cell discrimination dye (ViViD), we saw that either of them did not affect viability suggesting that they are non-toxic to cells.

In summary all our results explain that even after binding with MNP, BDNF retains its activity and it is as effective as free form of BDNF. This support our hypothesis that MNP bound BDNF can be used as therapeutic approach in drug abused population. Through the use of an in vitro BBB model, this study has established fundamental observation regarding the neuroprotective activity of MNP bound BDNF.

Future studies will investigate whether MNP-BDNF is neuro-protective in vivo in animal models. Studies are also under investigation for simultaneous binding of HIV drug and BDNF with magnetic nanocarriers, which will help in targeting HIV infection and drug abuse related issues simultaneously.
